# Reemergence of yellow fever in the state of São Paulo: the structuring role of surveillance of epizootics in non-human primates in a one health approach

**DOI:** 10.1590/1980-549720240064

**Published:** 2024-12-16

**Authors:** Leila del Castillo Saad, Francisco Chiaravalloti-Neto

**Affiliations:** I Universidade de São Paulo, School of Public Health – São Paulo (SP), Brazil.

**Keywords:** Yellow fever, Primates, Epidemiologic surveillance, One health

## Abstract

**Objective:**

to present a comprehensive analysis of YF occurrence of in the state of São Paulo since its reemergence, and the ongoing process of structuring the surveillance of epizootics in non-human primates in a one health approach.

**Methods:**

descriptive study of human cases and epizootics in non-human primates, structuring actions and the one health approach used in the state of São Paulo for yellow fever surveillance from 2000 to 2023.

**Results:**

from 2000 to 2023, 679 human cases and 857 epizootics in NHPs confirmed for yellow fever were recorded. There was an intensification of epizootic surveillance actions in non-human primates from 2017, when the virus circulated in areas without vaccine recommendations in the state.

**Conclusion:**

Five outbreaks were registered during the evaluated period, and the surveillance of epizootics in non-human primates played a fundamental role in implementing disease prevention and control measures in areas without vaccination recommendation, guiding vaccination actions, and serving as an example of how a one health approach can be adopted within epidemiological surveillance, making it more resilient to emerging threats.

## INTRODUCTION

More than two decades have passed since yellow fever (YF) was once again detected in the state of São Paulo (SP)^1^. This vector-borne, vaccine-preventable viral disease has a significant impact wherever it emerges^2,3^. In Brazil, two patterns of occurrence are recognized: sylvatic yellow fever (SYF) and urban yellow fever (UYF)^1-3^. Numerous reports emphasize the critical importance of YF in the context of surveillance and control, given its high lethality in severe cases and its considerable potential for dissemination^1-4^. Although the urban cycle has not been observed in Brazil since 1942, the reurbanization of YF remains a major concern, as its urban vector, *Aedes aegypti*, is widespread in nearly all densely populated urban areas of the country^5-7^.

YF is classified as a reemerging zoonosis. Of the pathogens identified as emerging or reemerging, 75% are of zoonotic origin^6,7^. The health of humans, animals, and the environment are interconnected and inseparable, a concept that forms the basis of the One Health approach. This paradigm, which has gained prominence in recent years, plays a crucial role in discussions on expanded health across various contexts in which it is observed^6,7^.

After nearly 50 years of epidemiological silence, the state of São Paulo began detecting the reemergence of wild circulation of the yellow fever virus within its borders in 2000^1^. In 2003, the state's Epidemiological Surveillance Center (*Centro de Vigilância Epidemiológica* – CVE), in collaboration with the Ministry of Health (MoH) and municipalities, initiated a surveillance program for epizootics in non-human primates (NHPs). This effort aimed at early detection of viral circulation within its enzootic cycle, utilizing a One Health approach to facilitate prevention and control measures for YF. Since then, numerous training sessions, meetings, and qualifications have been conducted to enhance and improve epidemiological data on the presence or absence of the virus through NHP surveillance^1^.

YF continued to exhibit non-endemic behavior, making it challenging to predict its circulation patterns. Consequently, the state was divided into areas with vaccine recommendation (AWVR), which indicated evidence of viral circulation in vectors, NHPs, or humans, and areas without vaccine recommendation (AW/oVR). This classification remained in place until 2019, when the entire state was designated as AWVR^2,8,9^.

Since 2016^2,7,8^, the state has experienced the highest level of viral circulation in its territory since the major UYF epidemics of the 19^th^ century^9^. In 2017, when YF reached populated regions previously classified as AW/oVR, epizootic surveillance in NHPs was essential not only for confirming the enzootic circulation of the virus but also for coordinating, designing, and planning vaccination efforts^9,10^.

During the largest outbreak of viral circulation in the state of São Paulo in recent history, the yellow fever virus spread rapidly to areas where vaccination was not previously recommended. This situation necessitated the immunization of a large segment of the population within a short timeframe. Consequently, the state revised its existing approach, which was based on the Ministry of Health's strategy of vaccinating in affected areas and surrounding regions. The new methodology focused on vaccination through ecological corridors of viral dispersion, utilizing data generated from epizootic surveillance in NHPs^2,4,9^.

This study aimed to describe, according to time, place, person, and NHP, the occurrence of YF in the state of SP since its reemergence, which began in 2000, as well as the ongoing process of structuring surveillance in NHPs. It will also identify and discuss the implications of these developments in the context of actions recommended for the prevention and control of the disease, and how the One Health approach has been essential in this context and should be expanded in the epidemiological methods applied to epidemiological surveillance.

## METHODS

This descriptive study utilizes secondary data from the Notifiable Diseases Information System (*Sistema de Informação de Agravos de Notificação* – SINAN), specifically from the modules on YF and epizootics in NHPs. The data includes information on epidemiological investigations and technical reports developed by state and municipal surveillance technicians regarding autochthonous human cases and confirmed epizootics in NHPs from 2000 to 2023. This information is available through the Division of Vector-Borne Diseases and Zoonoses (DVZOO) of the São Paulo State Health Secretariat (SES) and spans two decades.

YF surveillance is conducted through notifications of suspected occurrences in both human cases and epizootics in NHPs; the definitions of suspected and confirmed cases are outlined in Chart 1.

**Chart 1:** Definitions applied by epidemiological surveillance for yellow fever. Brazil, 2023.

Components	Suspicion definitions	Confirmed cases
Humans	Patient with acute fever (up to seven days) of sudden onset, with jaundice, coming from a risk area for yellow fever or locations with occurrences of epizootics in non-human primates or virus isolation in vectors in the last 15 days, without proof of vaccination against yellow fever (presentation of vaccination card).	Clinical-laboratory criterion Suspected case presenting at least one of the following conditions: Isolation of the yellow fever virus. Detection of viral genome. In atypical situations and/or detection of isolated events in time and space. In cases of epidemiological relevance, detection of viral genome fragments must be accompanied by clinical, epidemiological, and laboratory findings, and, if necessary, followed by genetic sequencing. Detection of IgM antibodies by the ELISA technique in unvaccinated individuals, associated with clinical, epidemiological, and laboratory findings. Fourfold or greater increase in antibody titers detected in serology from paired samples by the hemagglutination inhibition (HI) technique. Histopathological findings showing lesions compatible with recent yellow fever infection in eligible diagnostic tissues accompanied by the detection of viral antigens by immunohistochemical techniques. An asymptomatic or oligosymptomatic individual originating from active search and unvaccinated against yellow fever, who tested positive through a conclusive laboratory technique, may be considered a confirmed case. Epidemiological link criterion Suspected yellow fever case that progressed to death within ten days, without laboratory confirmation, during a period and in an area compatible with an outbreak or epidemic, where other cases and/or epizootics of NHPs have already been confirmed.
Epizootics in NHPs	Non-human primate of any species found dead (including remains) or sick in any location in the national territory.	By Laboratory: Conclusive laboratory result in at least one animal. By Epidemiological Link: Epizootic in primate associated with evidence of viral circulation in mosquitoes, other primates, and/or human cases in the probable infection site. Time and area of detection must be considered, evaluated on a case-by-case basis in conjunction with the State Health Secretariat and the Health Surveillance Secretariat.

NHP: non-human primates.

Source: Developed by the author. Brazil, 2023.

The study site was the state of SP, which comprises 645 municipalities covering an area of 248,221.996 km² and an estimated population of 44,420,459 inhabitants, according to 2022 data from the Brazilian Institute of Geography and Statistics (*Instituto Brasileiro de Geografia e Estatística* – IBGE). Of the 645 municipalities, 509 have populations of fewer than 50,000 inhabitants. Despite their large number, these municipalities account for only 16% of the state's total population. Data from the 2022 census indicated that 1,676,948 people live in rural areas, highlighting the significant urbanization of São Paulo^11^.

All autochthonous human cases and confirmed epizootics in NHPs reported to the state of SP YF surveillance system from January 2000 to December 2023 were included in the study. The following variables were analyzed for human cases: date of symptom onset (month and year), gender, median age, lethality, and the municipality of the probable site of infection. For epizootics in NHPs, the variables analyzed were: date of occurrence, gender of the NHPs, and municipality of occurrence.

For over 20 years, the CVE DVZOO has coordinated efforts to implement and improve this component of disease surveillance in the state, including training, qualifications, and technical meetings with municipalities. Epidemiological data from disease surveillance were used to identify municipalities with viral circulation and to describe human cases and epizootics in NHPs. Additionally, technical documents available at the CVE DVZOO were utilized to present the continuous process of structuring surveillance in NHPs.

## RESULTS

A total of 679 autochthonous human cases and 857 confirmed epizootics in NHPs for yellow fever have been recorded by the SES since the reemergence of the YF virus in 2000. During this period, five outbreaks were reported: in 2000, 2008, 2009, from 2016 to 2020, and in 2022 and 2023. The overall lethality rate was 35.7%, with a median age of 43.5 years. Men were predominantly affected, accounting for 73.7% of the cases (Table 1). The areas where viral circulation was detected expanded progressively over the more than 20-year period, increasing from two municipalities in 2000 to 140 in 2023, representing a 22% increase relative to the total number of municipalities in São Paulo.

**Table 1:** Characteristics of confirmed cases of yellow fever in humans (n=679) and non-human primates (n=857). São Paulo, 2000–2023.

Years	Human cases					Epizootics in non-human primates					
	Cases		Deaths		Fatality rate (%)	No. of positive epizootics in NHPs	No. of positive epizootics in NHPs with gender identification	Identified gender of NHPs			
	M	F	M	F				*Callithrix* spp	*Alouatta* spp	*Cebus* spp	*Callicebus* spp
2000	2	—	2	—	100	—	—	—	—	—	—
2008	2	—	2	—	100	4	4	—	4	—	—
2009	18	10	7	4	39.3	2	2	—	2	—	—
2016	2	1	2	1	100	32	32	5	22	5	—
2017	65	10	35	3	50.7	553	428	34	376	9	9
2018	403	96	152	21	34.7	244	190	32	147	5	6
2019	61	6	13	—	19.4	19	18	3	14	1	
2020	—	—	—	—	—	2	2	2	—	—	—
2022	1	—	—	—	—	—	—	—	—	—	—
2023	2	—	1	—	50	1	1	—	—	—	1

NHP: non-human primates.

Note: In the years 2001–2007, 2010, 2015, and 2021, no human cases or epizootics were confirmed.

**Figure 1:**
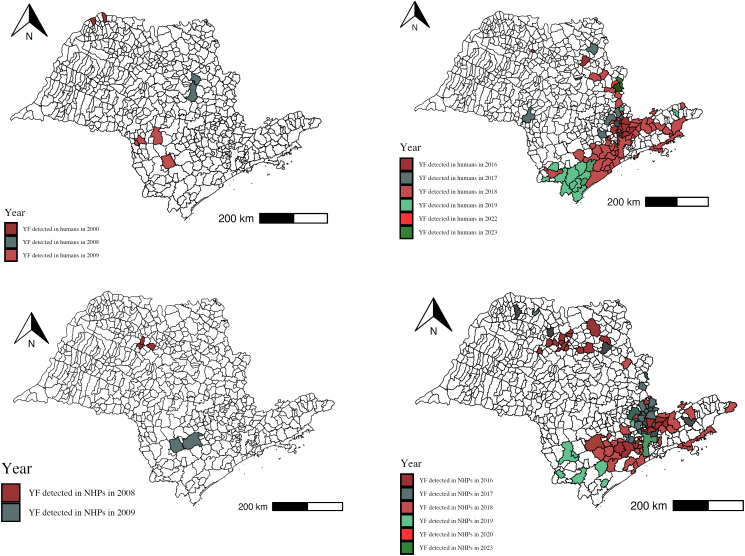
A) Municipalities with confirmed human cases of yellow fever in the state of São Paulo, from 2000 to 2023; B) Municipalities with confirmed epizootics in non-human primates for yellow fever in the state of São Paulo, from 2008 to 2023.

**Figure 2:**
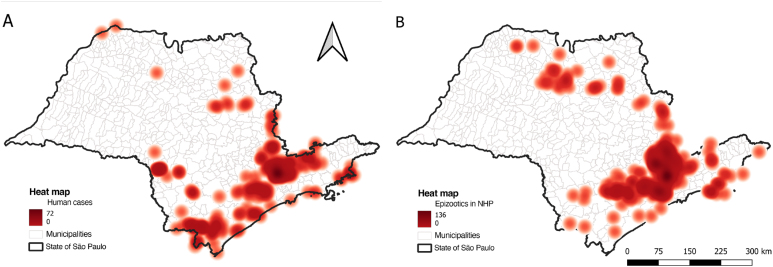
A) Heat map of confirmed human cases of yellow fever in the state of São Paulo, from 2000 to 2023; B) Heat map of confirmed epizootics in non-human primates for yellow fever in the state of São Paulo, from 2008 to 2023.

Surveillance of epizootics in NHPs that tested positive for YF has confirmed 857 events since 2003. Of these, 677 (79%) identified the genus of the affected primate, with a predominance of *Alouatta sp* (565, 83.4%) (Table 1). Initial confirmations of NHP deaths due to YF occurred in 2008 and 2009 in the regions of São José do Rio Preto and Botucatu, respectively, with subsequent occurrences in 2016, following the detection of a human case (fatality) also in the São José do Rio Preto region (Table 1 and Figures 1 and 2).

**Figure 3:**
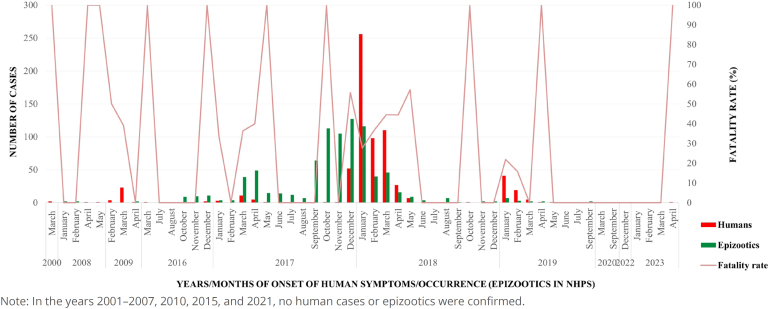
Historical series of confirmed human cases, lethality, and epizootics in non-human primates for yellow fever in the state of São Paulo, from 2000 to 2023.

Note: In the years 2001–2007, 2010, 2015, and 2021, no human cases or epizootics were confirmed.

The successive transmissions of yellow fever from 2016 to 2019 accounted for approximately 94.8% of confirmed human cases. During this period, there was a marked increase in the surveillance of epizootics in NHPs, with 851 confirmed epizootic events between 2016 and 2020. This represents 99.3% of the total cases in the historical series analyzed (Table 1 and Figures 1 and 3).

The peak of yellow fever transmission occurred at the turn of 2017 to 2018, with over 350 confirmed events. The detection of epizootics in NHPs intensified in September 2017, reaching 127 recorded events by December. Human cases peaked in January and February 2018, with over 300 confirmed cases, with the municipality of Mairiporã identified as the likely site of infection for 181 of these cases (Figures 1, 2, and 3).

The virus continued its spread toward the southern region of the state, reaching the Ribeira Valley by the end of 2018, with the identification of 67 human cases and a few confirmed epizootic events in NHPs, with the lowest lethality rate of the entire transmission period (19.4%) at the beginning of 2019 (Figures 1, 2 and 3).

Since 2017, following the detection of human and NHP cases in 2016, a technical group was formed comprising professionals from SES, the Secretariat of the Environment (*Secretaria de Meio Ambiente* – SMA), now the Secretariat of Infrastructure and Environment (*Secretaria de Infraestrutura e Meio Ambiente* – SIMA), and the Department of Wildlife (*Departamento de Fauna* – DeFau), which coordinates *ex situ* and *in situ* Wildlife Management Centers. Additionally, the São Paulo Pro-Primate Commission, also linked to SMA, the Military Environmental Police, and the Technical Commission for Veterinary Public Health, composed of members from the Regional Council of Veterinary Medicine of São Paulo, participated in this group. In collaboration, these institutions developed several alerts, training sessions, and guidelines aimed at expanding the network of veterinarians, biologists, and zootechnicians, among other professionals. Their efforts focused on addressing events involving the death and/or illness of primates in SP, thereby improving the quality of information generated from NHP surveillance.

After the virus spread to the southern part of the state, two epizootic events in NHPs were detected in 2020, in the municipalities of Barueri and São José do Rio Preto. The most recent yellow fever circulation events during the period analyzed occurred in 2023, with confirmed epizootics in NHPs and human cases in the São João da Boa Vista region (Table 1 and Figures 1 and 3).

## DISCUSSION

The data presented here must be analyzed from a different perspective, considering advances in scientific knowledge and the surveillance tools available during each transmission period. In 2000, molecular biology diagnostic tools were not as widely used, applied, or sensitive as they are today, which limited the methods for confirming and investigating suspected cases at that time^1,8^. Additionally, epizootic surveillance in NHPs was not yet a standard practice in disease surveillance and was only implemented in 2003. It was not until detections outside the vaccination recommendation areas in 2017 that this method was recognized as a robust tool for detecting viral circulation^1,2,12-16^.

Significant effort and coordination were undertaken by state surveillance to assist municipalities in the timely collection and reporting of events. This discussion occurred within the framework of the Brazilian Unified Health System (*Sistema Único de Saúde* – SUS), specifically in the meetings of regional bipartite inter-management councils. During these meetings, municipalities deliberated on mutual aid actions to support the collection of samples from NHPs.

Over more than two decades of analysis in this study, YF has demonstrated unpredictable behavior in its spatial reemergence^1,12-15,17^, influenced by the context and routes of national dispersion that the virus has followed^3,13^. Since 2017, a discernible trend of dispersion has been observed, particularly as the virus reached the Atlantic Forest and Serra do Mar regions in SP^2,13,14^.

The endemicity suggested during the intense transmission of YF in the state since 2016 does not appear to have materialized. The unpredictability of its occurrence, influenced by numerous known and unknown factors favoring transmission, indicates a contrary scenario^18^. There remain significant gaps in understanding the transmission mechanisms, processes leading to outbreaks, and the dispersion and persistence of the virus in nature^18,19^.

Given the unpredictability of identifying locations where YF reemerges^13,14,16,18,19^ and its significant epidemiological importance, disease surveillance must be conducted sensitively, particularly through epizootic surveillance in NHPs^10,14,16^. Strategies have been implemented to enhance engagement and sensitivity in NHP surveillance, involving collaboration among various state agencies within and outside the health sector. This has led to the establishment of an expanded NHP surveillance network, which includes key contributions from SIMA, the Environmental Military Police, universities, professional councils, independent veterinarians, and non-governmental organizations, among others.

In the historical series analyzed, 79% (n=677) of the epizootics in NHPs confirmed for yellow fever had gender identification, with monkeys of the species *Alouatta spp* and *Callithrix spp* being the most frequently infected. Few occurrences were recorded in species of the genera *Cebus spp* and *Callicebus spp*. These results align with previous findings, confirming that *Alouatta spp* and *Callithrix spp* are more frequently affected by the disease and are more commonly recorded than other species present in the state of SP^8,13,15,19^.

Other states affected by yellow fever have struggled to produce qualified information regarding the occurrence of the disease in NHPs. Similar studies indicated that only 10% of confirmed epizootics were identified by gender^12-14^. During the outbreak from 2016 to 2018, Minas Gerais confirmed 1,006 human cases and 448 epizootics in NHPs^13^. The occurrence of more human cases following the detection of circulation in NHPs underscores the essential role of NHP surveillance in monitoring yellow fever by detecting viral circulation while it remains in its enzootic cycle^3,4,12^. Therefore, the strategy adopted by the state of SP was successful, facilitating the vaccination of susceptible populations and recording a lower lethality rate than that reported by the Ministry of Health^2,4,9^.

The collaborative, intersectoral, multidisciplinary, networked action, with a broader perspective within the One Health framework, facilitated better data qualification by providing georeferenced information from epizootic surveillance in NHPs. This data was instrumental in developing a new vaccination strategy based on the methodology of ecological corridors of viral dispersion, particularly in a context of national shortages of immunobiologicals due to YF circulation in several Brazilian states^2,9,14,16^. Such information led to significant scientific contributions, enhancing not only YF surveillance in NHPs but also the conservation of these animals^15,16^, thereby supporting a shift away from an anthropocentric paradigm of surveillance.

When comparing information from animal surveillance with that from investigations into human cases, where surveillance practices have often been limited to merely confirming/discarding cases, frequently without assessing the probable site of infection, it becomes evident that data from animal surveillance can provide a more comprehensive understanding of the phenomenon. This is particularly relevant given the occurrence of the disease in wild conditions over the more than 20 years of re-emergence^1,2,13,14,16,17^.

It is understandable that municipalities struggle to improve the quality of information, given the successive cuts and dismantling that SUS has faced over the years^20^. Moreover, this reflects a fragile understanding of the role of epidemiological surveillance and the health-disease model that has been applied, executed, and discussed^20,21^. It is important to systematically improve the quality of information to comprehend phenomena and, thus, expand the discussion of anthropocentric epidemiological surveillance^21,22^ to a One Health approach^21-23^.

Surveillance of epizootics in NHPs (and other animal diseases) is primarily conducted by veterinarians and biologists working within SUS. These professionals, who are already limited in municipal staffing, have become even scarcer following the federal funding cuts in 2019 that affected the operation of the Family Health Support Centers (*Núcleos de Apoio à Saúde da Família* – NASFs)^17,24^, which included provisions for veterinarians’ participation in primary care. These professionals understand the concept of One Health, as it is intrinsic to their activities. This understanding reflects a movement toward implementation, capillarization, and discussion in the daily routine of expanded actions, supported by the principles of comprehensiveness and multidisciplinarity within the health system^22-26^.

The incorporation of a perspective centered on the social determination of diseases^20^ places us in a paradigm of reflection-action in health that extends beyond human health (alone). It is essential and urgent to engage in discussions that encompass mechanisms within surveillance, facilitating not only the systematic and qualified collection of data but also the creation of dialogic and reflective spaces on the subject.

Although the concept of One Health is not new and has been central to interdisciplinary and multisectoral discussions for years, there is currently a growing interest in applying this approach and translating it into action. This trend reinforces its general objectives and, in particular, emphasizes ecohealth (highlighting the ecocentric *versus* anthropocentric scope) and planetary health, explicitly recognizing the importance of environmental and ecosystem health.

The limitations identified in this study are inherent to research utilizing secondary databases, specifically those derived from passive notification processes. These limitations can result in inaccuracies in applying case definitions, as well as in the suspicion and confirmation of suspected cases. Additionally, they can affect the notification, epidemiological investigation, and registration of cases within the system.

This study highlights the potential for systematically incorporating the One Health approach into epidemiological surveillance methods, thereby advancing a paradigm that expands the scope of anthropocentric surveillance.

During the period analyzed in this research, five outbreaks of yellow fever were recorded in the state of São Paulo, resulting in the confirmation of 679 autochthonous human cases and 857 epizootics in NHPs. The most severe transmission occurred since 2016, when the virus reached areas and populations that had (previously) not been included in vaccine recommendations, necessitating the expansion of animal surveillance strategies to inform decision-making. This successful strategy, proposed by the Ministry of Health since 2003, underscores the effectiveness of the One Health approach in actions undertaken at all three levels of SUS management, enhancing the system's resilience and sensitivity to the emergence/reemergence of zoonotic diseases.
