# Supplementary material for: Reemergence of yellow fever in the state of São Paulo: the structuring role of surveillance of epizootics in non-human primates in a one health approach
Source: Rev Bras Epidemiol. 2024 Dec 16;27:e240064. doi: 10.1590/1980-549720240064 (PMC11654290; doi:10.1590/1980-549720240064)
Supplement: Translation

# Translation: Reemergência da febre amarela no estado de São Paulo: o papel estruturante da vigilância de epizootias em primatas não humanos numa abordagem em saúde única

**DOI:** 10.1590/1980-549720240064.2

**Authors:** Leila del Castillo Saad, Francisco Chiaravalloti-Neto

**Affiliations:** I Universidade de São Paulo, Faculdade de Saúde Pública – São Paulo (SP), Brasil.

**Keywords:** Febre amarela, Primatas, Vigilância epidemiológica, Saúde única

## Abstract

**Objetivo:**

Apresentar uma análise abrangente da ocorrência da febre amarela (FA) no estado de São Paulo desde a sua reemergência e o processo contínuo de estruturação da vigilância de epizootias em primatas não humanos (PNHs) numa abordagem em saúde única.

**Métodos:**

Estudo descritivo dos casos humanos e de epizootias em PNHs, bem como das ações estruturantes e da abordagem em saúde única empregadas no estado de São Paulo para a vigilância da febre amarela de 2000 a 2023.

**Resultados:**

De 2000 a 2023, foram registrados 679 casos humanos e confirmadas 857 epizootias em PNHs para FA. Houve intensificação das ações de vigilância de epizootias em PNHs desde 2017, quando o vírus circulou em áreas sem recomendação de vacina no estado.

**Conclusão:**

Foram registrados cinco surtos no período avaliado, e a vigilância de epizootias em primatas não humanos desempenhou papel fundamental para a implementação de medidas de prevenção e controle da doença no estado, nas áreas sem recomendação de vacina, guiando as ações de imunização, e é exemplo de como a abordagem em saúde única pode ser adotada no âmbito da vigilância epidemiológica, tornando-a mais resiliente às ameaças emergentes.

## INTRODUÇÃO

Mais de duas décadas se passaram desde que a febre amarela (FA) foi novamente detectada no estado de São Paulo (ESP)^1^. Doença viral de transmissão vetorial e imunoprevenível, gera grande impacto onde quer que seja detectada^2,3^. No Brasil, são descritos dois padrões de ocorrência: a febre amarela silvestre (FAS) e a febre amarela urbana (FAU)^1-3^. Inúmeros relatos apresentam a FA como sendo de elevada importância no contexto da vigilância e do controle dada sua acentuada letalidade entre os casos graves da doença e seu grande potencial de disseminação^1-4^. Embora o ciclo urbano não ocorra no Brasil desde 1942, a reurbanização da FA é motivo de grande preocupação, visto que seu vetor urbano, o *Aedes aegypti*, apresenta-se disseminado em quase a totalidade das áreas urbanas densamente povoadas do país^5-7^.

A FA é considerada uma zoonose reemergente. Dos patógenos considerados emergentes ou reemergentes, 75% são de origem zoonótica^6,7^. Saúde humana, animal e ambiental são interdependentes e indissolúveis, cerne do conceito de saúde única, despontando há alguns anos como paradigma importante da discussão sobre saúde ampliada nos diversos contextos em que se apresenta^6,7^.

Depois de aproximadamente 50 anos de silêncio epidemiológico, o ESP passou a detectar a reemergência da circulação silvestre do vírus em suas fronteiras no ano 2000^1^. Em 2003, foram iniciadas pelo Centro de Vigilância Epidemiológica estadual (CVE), em parceria com o Ministério da Saúde (MS) e os municípios, ações de estruturação da vigilância de epizootias em primatas não humanos (PNHs), visando à detecção precoce da circulação viral, ainda em seu ciclo enzoótico, numa abordagem em saúde única, oportunizando ações de prevenção e controle da FA. Desde 2003, foram realizados diversos treinamentos, reuniões e capacitações, visando ampliar e qualificar a informação epidemiológica de presença ou ausência viral, por meio da vigilância de PNHs^1^.

A FA continuou a apresentar comportamento não endêmico, não sendo possível prever seu comportamento de circulação. Assim, o estado era dividido em áreas com recomendação de vacina (ACRV), isto é, com evidência de circulação viral, seja em vetores, PNHs ou humanos, e áreas sem recomendação de vacina (ASRV), até 2019, quando o estado inteiro passou a ser ACRV^2,8,9^.

O estado registrou, desde 2016^2,7,8^, a maior circulação viral em seu território desde as grandes epidemias de FAU, no século XIX^9^. Ainda em 2017, quando a FA atingiu regiões populosas, nas áreas anteriormente denominadas ASRV, a vigilância de epizootias em PNHs foi fundamental não só para a confirmação da circulação enzoótica do vírus, mas também para a articulação, o delineamento e o planejamento das ações de vacinação^9,10^.

Em meio ao maior surto na história recente de circulação viral no estado de São Paulo, o vírus expandiu-se rapidamente para áreas sem recomendação de vacina, sendo necessário imunizar um grande contingente populacional em curto período. Desta maneira, o estado alterou a proposta vigente até então, proveniente do Ministério da Saúde, que trabalhava com a vacinação em área afetada e área ampliada, para a metodology de vacinação percorrendo corredores ecológicos de dispersão viral, embasada nas informações geradas por meio da vigilância de epizootias em PNHs^2,4,9^.

Este estudo pretende descrever, segundo tempo, lugar, pessoa e PNH, a ocorrência da FA no ESP desde a sua reemergência, que se iniciou no ano 2000, bem como o processo contínuo de estruturação da vigilância em PNHs, e apontar e discutir seus desdobramentos no cenário das ações preconizadas na prevenção e no controle do agravo, além de discutir como a abordagem em saúde única foi essencial nesse contexto e deve ser ampliada no método epidemiológico aplicado à vigilância epidemiológica.

## MÉTODOS

Estudo descritivo utilizando dados secundários do Sistema de Informação de Agravos de Notificação (SINAN), dos módulos FA e epizootias em PNHs; informações referentes às investigações epidemiológicas e a relatórios técnicos desenvolvidos pelos técnicos das vigilâncias estaduais e municipais, de casos humanos autóctones e epizootias em PNHs confirmados para FA no estado desde 2000 até 2023, disponíveis na Divisão de Doenças Transmitidas por Vetores e Zoonoses (DVZOO) do CVE da Secretaria de Estado da Saúde de São Paulo (SES) ao longo de duas décadas.

A vigilância da FA é realizada por meio de notificações de suspeitas de ocorrência de casos humanos e epizootias em PNHs; as definições de casos suspeitos e confirmados estão apresentadas no Quadro 1.

**Quadro 1:** Definições aplicadas pela vigilância epidemiológica para febre amarela. Brasil, 2023.

Componentes	Definições de suspeição	Casos confirmados
Humanos	Paciente com febre aguda (de até sete dias), de início súbito, com icterícia, procedente de área de risco para febre amarela ou locais com ocorrência de epizootias em primatas não humanos ou isolamento de vírus em vetores nos últimos 15 dias, sem comprovação de ser vacinado contra febre amarela (apresentação do cartão de vacina).	Critério clínico-laboratorial Caso suspeito que apresente pelo menos uma das seguintes condições: Isolamento do vírus da febre amarela. Detecção do genoma viral. Em situações atípicas e/ou detecção de eventos isolados no tempo e no espaço. Em situações de relevância epidemiológica, a detecção de fragmento do genoma viral precisa ser acompanhada dos achados clínicos, epidemiológicos e laboratoriais e, se necessário ainda, deve ser seguida do sequenciamento genético. Detecção de anticorpos IgM pela técnica de ELISA em indivíduos não vacinados, associados aos achados clínicos, epidemiológicos e laboratoriais. Aumento de quatro vezes ou mais dos títulos de anticorpos detectados na sorologia em amostras pareadas pela técnica de inibição da hemaglutinação (IH). Achados histopatológicos que apresentem as lesões compatíveis com infecção recente por febre amarela nos tecidos elegíveis para o diagnóstico acompanhados da detecção de antígeno viral em técnica imuno-histoquímica. Pode ser considerado um caso confirmado aquele indivíduo assintomático ou oligossintomático que, originário de busca ativa e não vacinado contra febre amarela, apresentou resultado positivo por meio de técnica laboratorial conclusiva. Critério de vínculo epidemiológico Caso suspeito de febre amarela que evoluiu para óbito em menos de dez dias, sem confirmação laboratorial, em período e área compatíveis com surto ou epidemia, em que outros casos e/ou epizootias de PNHs já tenham sido confirmados laboratorialmente.
Epizootias em PNHs	Primata não humano de qualquer espécie encontrado morto (incluindo ossadas) ou doente em qualquer local do território nacional.	Por laboratório: resultado laboratorial conclusivo em pelo menos um animal. Por vínculo epidemiológico: epizootia em primata associada à evidência de circulação viral em mosquitos, outros primatas e/ou casos humanos no local provável de infecção. Devem ser considerados o tempo e a área de detecção, avaliando-se caso a caso, em conjunto com a Secretaria Estadual de Saúde e a Secretaria de Vigilância em Saúde.

PNH: primatas não humanos.

Fonte: Elaborado pela autora. Brasil, 2023.

O local de estudo foi o ESP, que possui 645 municípios distribuídos em 248.221,996 km^2^ e população estimada de 44.420.459 habitantes, conforme dados do Instituto Brasileiro de Geografia e Estatística (IBGE) em 2022. Dos 645 municípios, 509 apresentavam populações inferiores a 50 mil habitantes e, apesar de grande representatividade, concentravam apenas 16% da população do estado. Dados do censo de 2022 apontaram 1.676.948 habitantes vivendo no campo, população considerada rural, evidenciando a acentuada urbanização paulista^11^.

Foram incluídos todos os casos humanos autóctones e epizootias em PNHs confirmados, notificados ao sistema de vigilância de FA do ESP, no período de janeiro de 2000 a dezembro de 2023. Para os casos humanos, foram analisadas as seguintes variáveis: data de início dos sintomas (mês e ano), sexo e mediana de idade, letalidade e município do local provável de infecção. Para as epizootias em PNHs, foram analisadas as seguintes variáveis: data da ocorrência, gênero de PNHs e município de ocorrência.

A DVZOO do CVE coordena as ações referentes à implantação e à qualificação desse componente da vigilância do gravo no estado ao longo de mais de 20 anos, como treinamentos, capacitações e reuniões técnicas com os municípios. Para caracterizar os municípios com circulação do vírus, descrever os casos humanos e epizootias em PNHs, foram utilizados os dados epidemiológicos provenientes da vigilância do agravo. Para apresentar o processo de estruturação contínua da vigilância em PNHs, usaram-se documentos técnicos disponíveis na DVZOO do CVE.

## RESULTADOS

Foram incluídos 679 casos humanos autóctones e 857 epizootias em PNHs confirmados para febre amarela pela SES desde a reemergência do vírus da FA no ESP, no ano 2000. Durante esse período, foram registrados cinco surtos em 2000, 2008, 2009, de 2016 a 2020 e em 2022 e 2023, com letalidade global de 35,7% e mediana de idade de 43,5 anos. Os homens foram a população predominantemente atingida, representando 73,7% dos casos (Tabela 1). As áreas de detecção de circulação viral aumentaram progressivamente ao longo dos mais de 20 anos da série histórica apresentada, passando de dois municípios, em 2000, para 140, em 2023, representando o incremento de 22% em relação ao total de municípios paulistas.

**Tabela 1:** Características dos casos confirmados de febre amarela em humanos (n= 679) e em primatas não humanos (n= 857). São Paulo, 2000–2023.

Anos	Casos humanos					Epizootias em primatas não humanos					
	Casos		Óbitos		Letalidade (%)	N^o^ de epizootias em PNHs positivas	N^o^ de epizootias em PNHs com identificação de gênero	Gênero de PNHs identificados			
	M	F	M	F				*Callithrix* spp	*Alouatta* spp	*Cebus* spp	*Callicebus* spp
2000	2	—	2	—	100	—	—	—	—	—	—
2008	2	—	2	—	100	4	4	—	4	—	—
2009	18	10	7	4	39,3	2	2	—	2	—	—
2016	2	1	2	1	100	32	32	5	22	5	—
2017	65	10	35	3	50,7	553	428	34	376	9	9
2018	403	96	152	21	34,7	244	190	32	147	5	6
2019	61	6	13	—	19,4	19	18	3	14	1	
2020	—	—	—	—	—	2	2	2	—	—	—
2022	1	—	—	—	—	—	—	—	—	—	—
2023	2	—	1	—	50	1	1	—	—	—	1

PNH: primatas não humanos.

Nota: Nos anos 2001–2007, 2010, 2015 e 2021, não foram confirmados casos humanos ou epizootias.

A vigilância de epizootias em PNHs notificadas e positivas para FA evidenciam que, desde 2003, foram confirmados 857 eventos, 677 (79%) deles com identificação de gênero de primata acometido e o predomínio de *Aloutta sp* (565, 83,4%) (Tabela 1). Inicialmente, as mortes de PNHs em decorrência da FA foram confirmadas em 2008 e 2009, na região de São José do Rio Preto e Botucatu, respectivamente, voltando a ocorrer em 2016, posteriormente à detecção de um caso humano (óbito) também na região de São José do Rio Preto (Tabela 1 e Figuras 1 e 2).

**Figura 1:** A) Municípios com casos humanos confirmados de febre amarela no estado de São Paulo, de 2000 a 2023; B) Municípios com epizootias em primatas não humanos confirmadas para febre amarela no estado de São Paulo, de 2008 a 2023.

**Figura 2:** A) Mapa de calor de casos humanos confirmados de febre amarela no estado de São Paulo, de 2000 a 2023; B) Mapa de calor de epizootias em primatas não humanos confirmadas para febre amarela no estado de São Paulo, de 2008 a 2023.

As transmissões sucessivas de 2016 a 2019 foram responsáveis por aproximadamente 94,8% dos casos humanos positivos. Houve expressivo incremento na vigilância de epizootias em PNHs, entre 2016 e 2020, período em que foram confirmadas 851 epizootias em PNHs, representando 99,3% dos casos da série histórica estudada (Tabela 1 e Figuras 1 e 3).

**Figura 3:** Série histórica de casos humanos, letalidade e epizootias em primatas não humanos confirmados para febre amarela no estado de São Paulo, de 2000 a 2023.

Nota: Nos anos 2001–2007, 2010, 2015 e 2021 não foram confirmados casos humanos ou epizootias.

O pico máximo da transmissão ocorreu na virada de 2017 para 2018, com mais de 350 eventos confirmados. A detecção de epizootias em PNHs começou a intensificar-se em setembro de 2017, chegando a 127 eventos registrados em dezembro desse ano. Os casos humanos atingiram o ápice de detecção em janeiro e fevereiro de 2018, com mais de 300 casos confirmados, sendo o município de Mairiporã local provável de infecção de 181 registros (Figuras 1, 2 e 3).

O vírus persistiu avançando rumo ao sul do estado, chegando ao Vale do Ribeira no fim de 2018, com a identificação de 67 casos humanos e poucos eventos confirmados em PNHs, com a letalidade mais baixa de todo o período de transmissão (19,4%) no início de 2019 (Figuras 1, 2 e 3).

Desde 2017, na sequência da detecção dos casos humanos e em PNHs em 2016, formou-se um grupo técnico envolvendo profissionais da SES, da Secretaria de Meio Ambiente (SMA), atualmente Secretaria de Infraestrutura e Meio Ambiente (SIMA), e do Departamento de Fauna (DeFau), que coordena os Centros de Manejo de Fauna Silvestre *ex situ* e *in situ*, além da Comissão Pró-Primatas Paulista, também vinculada à SMA, da Polícia Militar Ambiental e da Comissão Técnica de Saúde Pública Veterinária, composta por membros do Conselho Regional de Medicina Veterinária de São Paulo. Essas instituições, em colaboração, elaboraram diversos alertas, treinamentos e orientações com o intuito de ampliar o alcance da rede de médicos-veterinários, biólogos e zootecnistas, entre outros profissionais, para os eventos envolvendo morte e/ou adoecimento de primatas no ESP, qualificando as informações provenientes de vigilância de PNHs.

Depois da expansão do vírus em sentido ao sul do estado, foram detectadas duas epizootias em PNHs em 2020 nos municípios de Barueri e São José do Rio Preto. Em 2023, foram detectados os últimos eventos de circulação da febre amarela no período analisado, com a confirmação de epizootia em PNHs e casos humanos na região de São João da Boa Vista (Tabela 1 e Figuras 1 e 3).

## DISCUSSÃO

É importante analisar os dados aqui apresentados de maneira distinta, à luz do conhecimento científico e usando ferramentas de vigilância disponíveis em cada momento de transmissão. Ainda no ano 2000, as ferramentas diagnósticas de biologia molecular não eram tão difundidas, aplicadas e sensíveis, assim, as maneiras de confirmação e investigation dos casos suspeitos eram limitadas^1,8^. Além desse aspecto, a vigilância de epizootias em PNHs ainda não era práxis inerente à vigilância do agravo, passando a ser implementada em 2003, e somente por meio de detecções fora da área de recomendação vacinal no estado ocorridas em 2017 é que foi preconizada como ferramenta robusta de detecção de circulação viral^1,2,12-16^.

Foi empreendido grande esforço e articulação da vigilância estadual para auxiliar municípios para a oportuna coleta e notificação dos eventos. Esse debate ocorreu nos espaços de pactuação do Sistema Único de Saúde (SUS), isto é, nas reuniões dos conselhos intergestores bipartites regionais, nas quais foram deliberadas ações de ajuda mútua dos municípios para apoiar a coleta das amostras provenientes de PNHs.

Ao longo de mais de duas décadas de análise deste estudo, a FA apresentou um comportamento imprevisível de sua reemergência espacial^1,12-15,17^, em decorrência do contexto e da influência da rota nacional de dispersão que o vírus apresentava^3,13^. Desde 2017, foi possível perceber a tendência de dispersão, quando o vírus atingiu a Mata Atlântica e a Serra do Mar no ESP^2,13,14^.

A endemicidade discutida e apresentada como provável ao longo da transmissão intensa de FA, detectada desde 2016 no estado, parece não ter se concretizado, visto que a imprevisibilidade de sua ocorrência, dentre os inúmeros fatores (conhecidos ou não) que propiciaram sua transmissão, parece evidenciar exatamente o contrário^18^. Ainda persistem lacunas referentes a mecanismos de transmissão, processos que levam a surtos, dispersão e persistência do vírus na natureza^18,19^.

Dada a imprevisibilidade de apontar os locais de reemergência da FA^13,14,16,18,19^ e sua inegável importância epidemiológica, deve-se manter a vigilância do agravo operando de forma sensível, por meio da vigilância de epizootias em PNHs^10,14,16^. Foram adotadas estratégias no sentido de ampliar o engajamento e a sensibilidade à vigilância de PNHs, para o que diversos órgãos estaduais intra e extrassaúde foram contatados, organizando-se em uma rede ampliada de vigilância de PNHs, com participação importante da SIMA, da Polícia Militar Ambiental, de universidades, conselhos de classe, médicos-veterinários autônomos e organizações não governamentais, entre outros organismos.

Na série histórica analisada, 79% (n=677) das epizootias em PNHs confirmadas para febre amarela têm identificação de gênero, sendo os macacos das espécies *Alouatta spp* e *Callithrix spp* mais frequentemente infectados. Poucas ocorrências foram registradas em espécies dos gêneros *Cebus spp* e *Callicebus spp*. Esses resultados são consistentes com achados anteriores e confirmam que as espécies *Alouatta spp* e *Callithrix spp* são mais frequentemente acometidas pela doença, e também registradas, do que outras espécies presentes no estado de São Paulo^8,13,15,19^.

Outros estados, também acometidos pela febre amarela, não conseguiram produzir informações tão qualificadas a respeito da ocorrência da doença em PNHs. Estudos semelhantes apontaram que 10% das epizootias confirmadas apresentavam identificação de gênero^12-14^. Minas Gerais, durante o surto de 2016 a 2018, confirmou 1.006 casos humanos e 448 epizootias em primatas não humanos^13^. A ocorrência de mais casos humanos diante da detecção da circulação em PNHs evidencia como a vigilância em PNHs é essencial para a vigilância de febre amarela ao detectar a circulação do vírus ainda em seu ciclo enzoótico^3,4,12^. Desta maneira, a estratégia adotada pelo ESP foi bem-sucedida, subsidiando a vacinação de populações suscetíveis, registrando-se também letalidade inferior à registrada pelo Ministério da Saúde^2,4,9^.

A atuação colaborativa, intersetorial, multidisciplinar, em rede, com olhar ampliado para o contexto da saúde única, permitiu melhor qualificação dos dados, fornecendo dados georreferenciados provenientes da vigilância de epizootias em PNHs, que foram utilizados para elaborar nova estratégia de vacinação baseada na metodology de corredores ecológicos de dispersão viral, num cenário de escassez nacional do imunobiológico diante da ocorrência de circulação da FA em diversos estados brasileiros^2,9,14,16^. Tais informações deram origem a importantes produções científicas, colaborando não só com a vigilância da FA em PNHs, mas também para conservação desses animais^15,16^, corroborando a mudança do paradigma da vigilância antropocentrada.

Quando as informações provenientes da vigilância animal são confrontadas com aquelas provenientes de investigações de casos humanos, em que a prática da vigilância tem-se limitado a confirmar/descartar o caso, muitas vezes, sem avaliar o local provável de infecção, é possível compreender melhor o fenômeno por meio dos dados provenientes da vigilância animal, também em função da ocorrência silvestre da doença nesses mais de 20 anos de reemergência^1,2,13,14,16,17^.

É compreensível que os municípios não consigam avançar na qualificação das informações, tendo em vista os sucessivos cortes e desmontes que o SUS vem sofrendo ao longo dos anos^20^, e mais do que isso: demonstra fragilidade de compreensão do papel da vigilância epidemiológica e de qual modelo de saúde-doença vem sendo aplicado, executado e discutido^20,21^. É importante qualificar as informações de maneira sistemática para compreender os fenômenos e, assim, ampliar o debate da vigilância epidemiológica antropocentrada^21,22^ para uma abordagem em saúde única^21-23^.

A vigilância de epizootias em PNHs (e outras doenças animais) é realizada, em grande parte, por médicos-veterinários e biólogos inseridos no SUS. Esses profissionais, ainda poucos nos quadros de servidores municipais, depois do corte do repasse federal, em 2019, para o funcionamento dos Núcleos de Apoio à Saúde da Família (NASFs)^17,24^, que previa a participação de médicos-veterinários na atenção primária, ficaram ainda mais escassos. Eles compreendem o conceito de saúde única, pois este é intrínseco às atividades que exercem, representando um movimento de implantação, capilarização e discussão no cotidiano do agir ampliado, apoiado no conceito de integralidade e multidisciplinaridade em saúde do sistema^22-26^.

A incorporação desse olhar, partindo da temática da determinação social das doenças^20^, coloca-nos num paradigma de reflexão-ação em saúde ampliada para além da saúde (apenas) humana. É importante e premente a discussão que permeie mecanismos, dentro da vigilância, e possibilite não só a coleta sistemática e qualificada de dados, mas espaços dialógicos e reflexivos sobre a temática.

Embora o conceito de saúde única não seja novo e esteja na vanguarda das discussões interdisciplinares e multissetoriais há anos, atualmente há interesse crescente para que essa abordagem seja aplicada e traduzida em ação, reforçando seus objetivos gerais e, particularmente, a ecossaúde (ao destacar o escopo ecocêntrico *versus* antropocêntrico) e a saúde planetária, reconhecendo explicitamente a relevância da saúde ambiental/ecossistêmica.

As limitações encontradas neste estudo são aquelas inerentes à pesquisa em bases de dados secundários, neste caso, procedentes dos processos de notificação passiva, o que pode levar a imprecisões na aplicação de definições de casos e na suspeição e na confirmação dos casos suspeitos, bem como sua notificação, investigation epidemiológica e registro no sistema.

O presente estudo apresenta o potencial de incorporação e qualificação sistemática da abordagem em saúde única para o método da vigilância epidemiológica, avançando num paradigma de ampliação de atuação de uma vigilância antropocentrada.

No período analisado nesta pesquisa, foram registrados cinco surtos de febre amarela no estado de São Paulo, com a confirmação de 679 casos humanos autóctones e 857 epizootias em PNHs, sendo a gravidade de maior magnitude a transmissão registrada desde 2016, quando o vírus atingiu áreas e populações (anteriormente) sem recomendação de vacina, fato que exigiu a ampliação de estratégias de vigilância animal para guiar a tomada de decisão. Essa estratégia bem-sucedida, proposta pelo Ministério da Saúde desde 2003, reforça como a abordagem em saúde única pode ser exitosa nas ações executadas nos três níveis de gestão do SUS, tornando o sistema mais resiliente e sensível à emergência/reemergência de doenças zoonóticas.
